# Childlessness and its associated factors among Chinese women: a nationwide population-based study

**DOI:** 10.1186/s12889-025-21829-x

**Published:** 2025-02-17

**Authors:** Qing Han, Junhua Dang, Yibo Wu, Wenting Ye

**Affiliations:** 1 Department of Social Policy and Intervention, University of Oxford, Oxford, UK; 2 Institute of Social Psychology, School of Humanities and Social Sciences, Xi’an Jiaotong University, Xi’an, China; 3 School of Public Health, Peking University, 38 Xueyuan Road, Haidian District, Beijing, 100191 China; 4 Faculty of Psychology, Southwest University (SWU), Beibei District, Chongqing, 400715 China; 5 Key Laboratory of Cognition and Personality (SWU), Ministry of Education, Chongqing, China

**Keywords:** Fertility, Childlessness, Reproductive anxiety, Women, Survey

## Abstract

**Background:**

China has experienced a continuous decline in fertility rates, despite the end of the one-child policy, further intensifying population ageing. Timely evidence on the factors associated with childlessness among Chinese women is needed, alongside examining reproductive anxiety among childless women of reproductive age.

**Methods:**

This nationwide, cross-sectional study was based on the 2023 *Psychology and Behavior Investigation of Chinese Residents* Survey. A total of 10,802 women across mainland China who were (or had been) married were included, of whom 5,885 were of reproductive age (20–49 years) and 4,917 of post-reproductive age (≥ 50 years). Logistic and linear regression analyses were conducted to examine factors associated with childlessness among reproductive-aged and post-reproductive women, and reproductive anxiety among childless women of reproductive age at intrapersonal, interpersonal, and societal levels, as outlined in the social-ecological model.

**Results:**

The comparison of reproductive patterns among women across different age groups showed a dramatic decline in the proportion of having three or more children, and a gradual increase in the childlessness over time. Among 5,885 women of reproductive age by the time of the survey, 612 (10.4%) reported having no children, while among 4,917 post-reproductive women, 161 (3.3%) were childless. Higher education level (OR = 2.83 [2.26, 3.55]; OR = 2.35 [1.44, 3.74]), being the only child in the family (OR = 2.85 [2.28, 3.55]; OR = 10.56 [7.38, 15.14]), and exposure to intimate partner violence (OR = 1.15 [1.02, 1.29]; OR = 1.68 [1.43, 1.98]) were significantly associated with childlessness among both reproductive-aged women and post-reproductive women. Poorer stress coping ability and exposure to sexual abuse during childhood were also associated with childlessness in post-reproductive women. Among childless women of reproductive age, higher education level, higher body image dissatisfaction, exposure to child abuse, parental issues during childhood, and belonging to ethnic minority groups were associated with increased reproductive anxiety.

**Conclusions:**

This study used the most recent nationwide survey data from China to identify factors associated with childlessness and reproductive anxiety, with an emphasis on socio-economic factors and domestic violence. The findings highlight the significant impact of domestic violence on fertility behaviors and reproductive anxiety, providing valuable evidence for future policy interventions.

**Supplementary Information:**

The online version contains supplementary material available at 10.1186/s12889-025-21829-x.

## Introduction

The decline in global fertility rates has garnered significant attention. As reported in the United Nations World Population Prospects (2022), the global total fertility rate (TFR) decreased from 2.58 children per woman in 2000 to 2.31 in 2020, approaching the replacement-level fertility threshold of 2.1. In China, the decrease has been even more dramatic, with the TFR dropping from 1.22 in 2000 to 1.07 in 2022 [1]. This decline in fertility rates exacerbates the issue of global population ageing [2, 3]. 

China is among the countries with a total fertility rate below the replacement level as of 2021^1^. The childlessness rate among women born between 1985 and 1989 has risen significantly, from 2.7% for those born in 1950 to 17.4% [4]. Population policies have significantly influenced China’s fertility rate. Improvements in health and education during the early socialist period laid the foundation for fertility decline [5–7]. 

Since 1963, the fertility rate has gradually declined, a change resulting from a complex interplay of urbanization, economic and social influences [6, 8, 9]. Throughout this period, China’s fertility policies have evolved through three stages, each aimed at regulating population size in accordance with material production and the capacity for population self-sustenance. In 1979, China implemented the “one-child” policy, which restricted most families to having only one child [10]. Since 2011, China has gradually relaxed its birth control policies, beginning with the “two-child policy for dual only-child parents,” which allowed couples, each of whom was the only child in their respective family, to have two children. In 2015, the policy was further revised to a universal “two-child policy,” allowing all couples to have two children, regardless of their family background. In 2021, the “three-child” policy was implemented, lifting restrictions on the number of children and introducing measures to encourage families to have three or more children. Despite the gradual relaxation of fertility policies, China’s fertility rate has not shown any significant increase [10, 11]. 

In recent decades, the dynamics of fertility and family planning have undergone significant transformations globally, including a trend toward delayed motherhood and a decline in the number of children born per family [1]. One of the most striking manifestations of these trends is the increasing proportion of childlessness observed in many countries, such as those in Europe and the United States [12–15]. There has also been a significant increase in the proportion of childlessness in China from 2010 to 2020 [16]. The factors contributing to childlessness are multifaceted, encompassing political, economic, social, health-related and psychological dimensions [17]. Given the rapid changes in the population structure and fertility patterns in China, more up-to-date evidence of the factors associated with childlessness and reproductive anxiety is needed.

The present study aims to examine the reproductive patterns among women in China, with a focus on the personal and social factors associated with childlessness (defined in this study as the current state of having no children) among women currently of reproductive age. This study is based on the 2023 Psychology and Behavior Investigation of Chinese Residents (PBICR) survey [18, 19, 20], a large-scale survey with strong population representativeness. While this study follows the WHO definition of reproductive age (15–49 years), the analysis was limited to women aged 20–49 due to constraints in the available data. The PBICR survey specifically targeted the reproductive status of women who are currently married or have been married; given that China’s legal minimum marriage age is 20 years, only women aged over 20 years completed the reproductive module.

Drawing on Bronfenbrenner’s (1979) social-ecological model, this study explores the factors associated with childlessness among women across intrapersonal, interpersonal, and societal levels. The model offers a holistic approach by capturing the dynamic interactions between individual, social, and environmental factors, providing a comprehensive framework for understanding the complexity of reproductive behaviors. Its application allows for the identification of multiple, intersecting influences that shape childlessness, which is crucial for informing targeted interventions [21, 22]. The study further examines the factors associated with reproductive anxiety among childless women of reproductive age. Focusing on reproductive anxiety among currently childless women allows us to explore the psychological factors that influence their decision not to have children, while also providing insights into potential future fertility prospects.

In the framework of the social-ecological model used in this study, beyond demographic and sociological factors, several key psychosocial factors that may influence fertility are examined. These include resilience, body image dissatisfaction, childhood trauma, and intimate partner relationships, among others. The particular significance of these factors lies in their modifiability—meaning they are not fixed but can be intervened upon, thus offering potential pathways for promoting positive reproductive outcomes. To the best of our knowledge, this is the first large-scale, population-based study on the personal and social factors associated with childlessness in China. In the context of China’s ageing population, this study provides a social-psychological understanding of the complex fertility dynamics and quantitative evidence for the formulation of policies aimed at increasing fertility rates.

## Methods

### Data source

The data for this study were obtained from the 2023 Psychology and Behavior Investigation of Chinese Residents survey, a nationwide cross-sectional survey conducted from June 20 to August 31, 2023, using multistage stratified and quota sampling methods. The survey covered 23 provinces and five autonomous regions, with 2–13 cities selected from each based on population size, and included four municipalities, Hong Kong, and Macao, totaling 150 cities. In the second stage, 800 communities or villages were randomly selected, with quotas for gender and age based on the 2021 Seventh National Population Census to ensure a balanced gender ratio and age distribution reflective of China’s population pyramid.

The study was approved by the Biomedical Ethics Committee of Shandong Provincial Hospital (No.: SWYX: NO.2023 − 198) and officially registered as an observational study in the China Clinical Trials Registry (Registration No.: ChiCTR2300072573; Registration date: June 16, 2023) [18, 19, 20]. 

### Participants

Eligible participants of the 2023 PBICR survey were required to be Chinese permanent residents, at least 18 years old, and capable of understanding and completing each item in the questionnaire independently or with the assistance of an investigator. Exclusion criteria included conditions such as confusion, psychiatric abnormalities, or cognitive impairment that would prevent participants from completing the survey.

In the present study, we focused on data from mainland China. Among the 14,946 female participants from 23 provinces, five autonomous regions and four municipalities in mainland China, 10,802 women who were (or had been) married and provided valid reproductive data were included in the analyses.

### Measurements

The 2023 PBICR survey collected data on the reproductive status with the question “How many children do you have?” The response options included “0”, “1”, “2”, and “≥3”. In this study, a reported number of zero children was considered as childlessness. Data on reproductive anxiety were recorded with the question “How anxious are you about (re)producing (i.e., your anxiety level that arises from difficulties in reproductive decision-making)?” on a visual analogue scale from 0 (not anxious) to 100 (very anxious). There were no missing values for the variables.

Potential factors were incorporated at three levels within the social-ecological system framework [21, 22]. Intrapersonal-level factors included educational level, stress coping (5-item scale), body image dissatisfaction (5-item scale), risk-taking (8-item scale), and subjective philanthropy (8-item scale). Interpersonal-level factors included whether the individual is the only child in their family, family property ownership, family support (5-item scale), exposure to child physical and psychological abuse (4-item scale), exposure to child sexual abuse (4-item scale), exposure to parental issues during childhood (e.g., addicted or depressed parents, 5-item scale), witnessing intimate partner violence (IPV) against the mother during childhood (4-item scale), self-experience of IPV (physical, sexual and psychological, 5-item scale), positive family atmosphere (4-item scale), having health insurance, and family social status (7-point scale). The societal-level factors were whether the individual belongs to a minority ethnic group or the Han ethnic group, and whether their household was registered as agricultural population or non- agricultural population. Complete measurement scales for these factors are provided in the Appendix A [18, 19, 20]. 

### Descriptive analysis of changes in reproductive patterns and policy shifts

The investigation of changes in reproductive patterns among Chinese women inevitably involves considering the shifts in family planning policies over time. The sample was divided into six groups based on their exposure to reproductive policies during their peak reproductive years (i.e., ages 20–35).

- Group 1: Women whose peak reproductive years occurred entirely before the initiation of the one-child policy in 1979 (i.e., aged over 35 in 1979).
- Group 2: Women who experienced the initiation of the one-child policy during their peak reproductive years (e.g., 25 years old in 1979).
- Group 3: Women whose peak reproductive years occurred entirely during the one-child policy era spanning from 1979 to 2011 (e.g., 18 years old in 1979).
- Group 4: Women who experienced the transition from the one-child policy to the two-child policy but not the three-child policy during their peak reproductive years (e.g., a woman aged 30 in 2011 when the two-child policy was initiated, and aged 40 in 2021 when the three-child policy was implemented).
- Group 5: Women who experienced the one-child policy, the two-child policy, and the three-child policy during their peak reproductive years (e.g., a women aged 21 in 2011 and 31 years in 2021).
- Group 6: Women whose peak reproductive years occurred entirely after the end of the one-child policy and experienced the two-child and three-child policies (i.e., less than 20 years old in 2011).

We used the data from these six groups to describe changes in the number of children among Chinese women, from before 1979 (prior to the implementation of the one-child policy) to the present. Through this analysis, the longitudinal trends in family structure changes in China, in response to evolving fertility policies, can be clearly demonstrated.

### Statistical analysis of influencing factors of childlessness and reproductive anxiety

Descriptive statistics for the three-level potential factors were presented for women currently of reproductive age, both overall and according to their childlessness status.

We employed binary logistic regression to examine potential factors associated with childlessness among women currently of reproductive age. This subset of participants was targeted, as they are the primary focus of population policies and family-planning intervention programs. The dependent variable was childlessness status (having children or not), with the three levels of factors as independent variables. Age (and age squared) and geographical location were controlled as covariates. In addition, we explored factors associated with childlessness among women of post-reproductive age following similar methods.

In the analysis of reproductive anxiety, we focused on the anxiety related to childlessness rather than anxiety about having another child. Thus, the analysis was restricted to childless women. We used linear regression to explore factors associated with reproductive anxiety among childless women of reproductive age, with reproductive anxiety as the dependent variable, and the same set of independent variables and covariates as in the analysis of childlessness status. Complete-case analyses were conducted for both logistic regression and linear regression due to the low proportion of missing values (3% and 5%, respectively). All analyses were conducted in R version 4.3.2 (R Core Team, 2023).

### Role of the funding source

The funders had no role in study design, data collection, data analysis, interpretation, or writing of the report.

## Results

### Participant characteristics and current childlessness situation

**Fig. 1:**
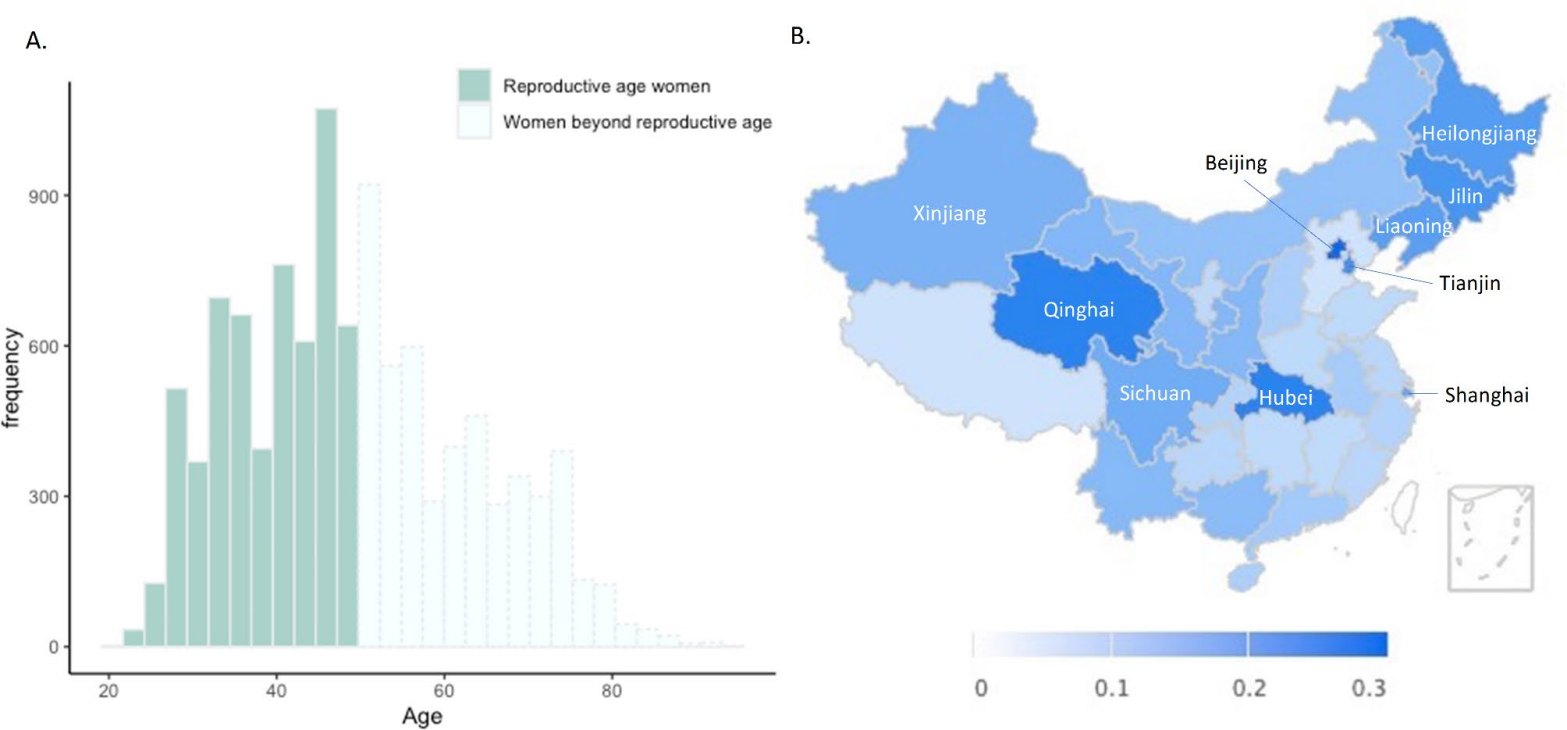
Age distribution of female participants and childlessness proportions of women across provinces in mainland China Note. Panel A illustrates the age distribution of women who were (or had been) married, where green bars represent those within the reproductive age, and light green bars indicate those post-reproductive age. Panel B displays the childlessness proportion among women of reproductive age across provinces/municipalities in mainland China. Provinces/municipalities with a childlessness proportion exceeding 15% are labelled with their names in the figure

A total of 10,802 women who were (or had been) married reported their reproductive status, with a mean age of 49.4 years (SD = 13.8). The age distribution of these participants is illustrated in Fig. 1 (Panel A). Within the sample, there were 5,885 women of reproductive age (20–49 years), among whom 612 (10.4%) reported having no children; 2,834 (48.2%) had one child; 2,103 (35.7%) had two children; and 336 (5.7%) had three or more children. In the post-reproductive group (≥ 50 years old), a total of 4,917 women were included, with 161 (3.3%) reporting childlessness; 1,734 (35.3%) had one child; 1,954 (39.7%) had two children; and 1,068 (21.7%) had three or more children.

The distribution of childlessness among women of reproductive age by province across mainland China is displayed in Fig. 1 (Panel B). To be noted, the regions with the highest proportions of childlessness in our sample generally correspond to regions with the lowest birth rates in China [23], suggesting good population representativeness. Furthermore, Tables 1 and 2 provide a descriptive summary of the three levels of potential factors within the social-ecological system framework, stratified by their childlessness status, among women of reproductive age and post-reproductive age, respectively.

**Table 1:** Descriptive statistics of factors at three levels within the social-ecological system framework in women of reproductive age

	Factors		Childless women (*N* = 612)	Women with child (*N* = 5,273)	Total (*N* = 5,885)
Intrapersonal level	Education level (Bachelor’s degree or above)	%	61.4%	31.5%	34.6%
	Stress coping	mean (SD)	3.66 (0.80)	3.70 (0.78)	3.70 (0.78)
	Body image dissatisfaction	mean (SD)	2.88 (1.01)	2.75 (0.98)	2.76 (0.98)
	Risk-taking	mean (SD)	3.03 (0.87)	2.90 (0.87)	2.91 (0.87)
	Subjective philanthropy	mean (SD)	3.80 (0.65)	3.78 (0.65)	3.78 (0.65)
Interpersonal level	The only child in the family	%	43.3%	16.9%	19.6%
	Family owns no property	%	13.3%	9.4%	9.8%
	Family support	mean (SD)	4.24 (0.68)	4.27 (0.65)	4.26 (0.65)
	Exposed to child abuse (physical and psychological)	%	15.5%	15.2%	15.2%
	Exposed to child abuse (sexual)	%	6.7%	4.4%	4.6%
	Exposed to parental issues during childhood	%	8.7%	7.2%	7.4%
	Witness of intimate partner violence during childhood	%	7.4%	5.8%	6.0%
	Intimate partner violence	mean (SD)	1.49 (0.93)	1.37 (0.76)	1.38 (0.78)
	Positive family atmosphere	mean (SD)	3.92 (0.78)	3.92 (0.75)	3.92(0.75)
	Have health insurance	%	96.2%	97.2%	97.1%
	Family social status	mean (SD)	4.09 (1.27)	4.00 (1.39)	4.01 (1.38)
Societal level	Ethnic minority	%	9.0%	7.3%	7.5%
	Household registration as agricultural population	%	46.1%	45.8%	45.8%

Note. SD = standard deviation

**Table 2:** Descriptive statistics of factors at three levels within the social-ecological system framework in women of post-reproductive age

	Factors		Childless women (*N* = 161)	Women with child (*N* = 4,756)	Total (*N* = 4,917)
Intrapersonal level	Education level (Bachelor’s degree or above)	%	19.9%	8.6%	9.0%
	Stress coping	mean (SD)	3.32 (0.86)	3.56 (0.79)	3.55 (0.80)
	Body image dissatisfaction	mean (SD)	2.75 (0.97)	2.43 (0.99)	2.44 (0.99)
	Risk-taking	mean (SD)	2.87 (0.84)	2.71 (0.90)	2.72 (0.90)
	Subjective philanthropy	mean (SD)	3.56 (0.79)	3.66 (0.67)	3.65 (0.67)
Interpersonal level	The only child in the family	%	50.3%	7.4%	8.8%
	Family owns no property	%	13.7%	6.0%	6.3%
	Family support	mean (SD)	3.66 (0.93)	4.13 (0.67)	4.11 (0.69)
	Exposed to child abuse (physical and psychological)	%	20.5%	18.5%	18.6%
	Exposed to child abuse (sexual)	%	13.0%	3.3%	3.6%
	Exposed to parental issues during childhood	%	16.8%	10.5%	10.7%
	Witness of intimate partner violence during childhood	%	14.9%	10.6%	10.8%
	Intimate partner violence	mean (SD)	2.08 (1.24)	1.38 (0.77)	1.40 (0.80)
	Positive family atmosphere	mean (SD)	3.65 (0.89)	3.90 (0.71)	3.89(0.72)
	Have health insurance	%	91.3%	96.9%	96.7%
	Family social status	mean (SD)	4.12 (1.47)	4.13 (1.31)	4.13 (1.32)
Societal level	Ethnic minority	%	15.5%	8.6%	8.9%
	Household registration as agricultural population	%	55.3%	59.1%	59.0%

Note. SD = standard deviation

### Changes in reproductive patterns among Chinese women over time and in response to policy shifts

**Fig. 2:**
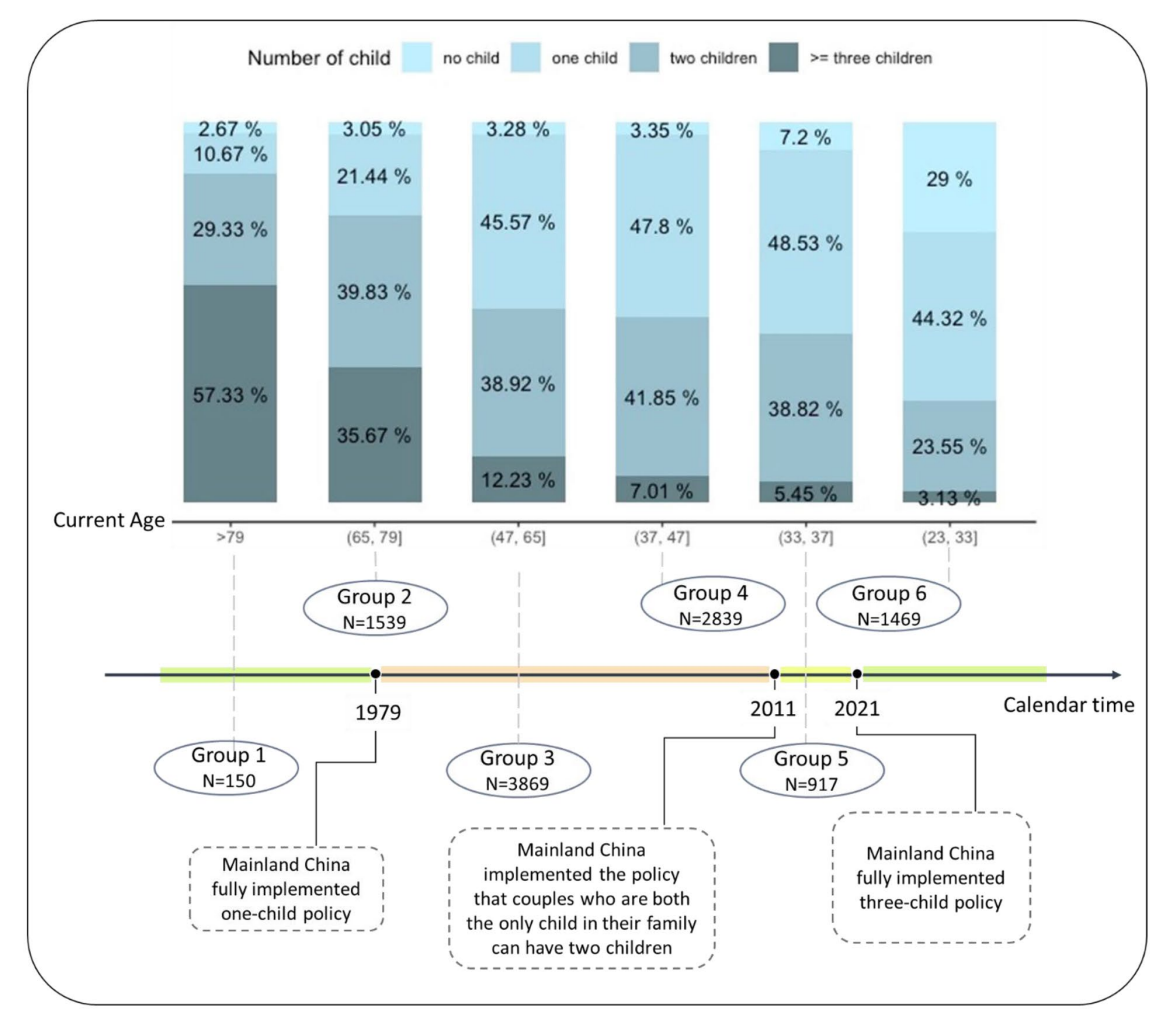
Three key family planning policy transition periods on the reproductive structure of Chinese women

As illustrated in Fig. 2, a comparison of reproductive structures across the six groups revealed a dramatic decline in the proportion of women having three or more children over time (from 57.33 to 3.13%; Fig. 2). Women whose peak reproductive years occurred during or after the implementation of the one-child policy (i.e., all groups except Group 1) predominantly shifted toward having one to two children, and eventually transitioned to mainly having only one child. In the meantime, the proportion of childless women was gradually increasing. It is worth noting that individuals in Groups 4–6 still have the potential to continue having children, especially in Group 6, hence making these groups less comparable to Groups 1–3.

### Factors associated with childlessness among women of reproductive age and post-reproductive age

As shown in Table 3, among women of reproductive age, those with a Bachelor’s degree or higher were significantly more likely to be childless, with an odds ratio (OR) of 2.83 (95% CI: 2.26, 3.55), compared to women with lower levels of education (*p* < 0.001). Additionally, women who were the only child in their family were 2.85 (95% CI: 2.28, 3.55) times more likely to be childless than those with siblings (*p* < 0.001). Exposure to IPV was also significantly associated with an increased likelihood of childlessness, with an OR of 1.15 (95% CI: 1.02, 1.29; *p* = 0.022). There was no evidence suggesting an association between other factors and childlessness in women of reproductive age (see Table 3).

**Table 3:** Factors associated with childlessness in women of reproductive age

	Factors	OR	95% CI	*p* value
Intrapersonal level	Education level (Bachelor’s degree or above)	2.83	2.26, 3.55	< 0.001
	Stress coping	0.87	0.74, 1.02	0.085
	Body image dissatisfaction	1.00	0.90, 1.12	0.941
	Risk-taking	1.14	1.00, 1.30	0.057
	Subjective philanthropy	1.03	0.86, 1.24	0.727
Interpersonal level	The only child in the family	2.85	2.28, 3.55	< 0.001
	Family owns no property	1.36	0.99, 1.87	0.055
	Family support	0.94	0.80, 1.12	0.506
	Exposed to child abuse (physical and psychological)	0.80	0.56, 1.11	0.185
	Exposed to child abuse (sexual)	1.00	0.62, 1.55	0.988
	Exposed to parental issues during childhood	1.15	0.76, 1.70	0.511
	Witness of intimate partner violence during childhood	1.31	0.81, 2.09	0.263
	Intimate partner violence	1.15	1.02, 1.29	0.022
	Positive family atmosphere	1.09	0.93, 1.27	0.289
	Have health insurance	0.83	0.47, 1.56	0.547
	Family social status	1.05	0.97, 1.13	0.243
Societal level	Ethnic minority	1.07	0.71, 1.58	0.739
	Household registration as agricultural population	1.25	1.00, 1.56	0.052

Note. OR = odds ratio; CI = confidence interval. All independent variables were entered into the same model to achieve mutual adjustment

As indicated in Table 4, women of post-reproductive age with a Bachelor’s degree or higher were more likely to be childless compared to their counterparts with lower educational levels (OR = 2.35, 95% CI: 1.44, 3.74; *p* < 0.001). Being the only child significantly increased the likelihood of childlessness, with an OR of 10.56 (95% CI: 7.38, 15.14; *p* < 0.001). Exposure to IPV also played a significant role, with women who had been exposed to IPV having greater odds of being childless (OR = 1.68, 95% CI: 1.43, 1.98; *p* < 0.001). Conversely, better stress coping abilities were associated with reduced likelihood of childlessness (OR: 0.76, 95% CI: 0.59, 0.97; *p* = 0.029). Additionally, exposure to sexual child abuse was associated with an increased likelihood of childlessness (OR: 2.10, 95% CI: 1.00, 4.17; *p* = 0.041). No evidence was found to support associations between other factors and childlessness in women of post-reproductive age (see Table 4).

**Table 4:** Factors associated with childlessness in women of post-reproductive age

	Factors	OR	95% CI	*p* value
Intrapersonal level	Education level (Bachelor’s degree or above)	2.35	1.44, 3.74	< 0.001
	Stress coping	0.76	0.59, 0.97	0.029
	Body image dissatisfaction	1.15	0.93, 1.41	0.191
	Risk-taking	1.06	0.84, 1.35	0.619
	Subjective philanthropy	0.95	0.71, 1.28	0.751
Interpersonal level	The only child in the family	10.56	7.38, 15.14	< 0.001
	Exposed to child abuse (physical and psychological)	0.66	0.35, 1.20	0.189
	Exposed to child abuse (sexual)	2.10	1.00, 4.17	0.041
	Exposed to parental issues during childhood	0.97	0.50, 1.79	0.928
	Witness of intimate partner violence during childhood	1.07	0.52, 2.14	0.846
	Intimate partner violence	1.68	1.43, 1.98	< 0.001
Societal level	Ethnic minority	1.49	0.85, 2.55	0.152
	Household registration as agricultural population	0.94	0.65, 1.37	0.744

Note. OR = odds ratio; CI = confidence interval. All independent variables were entered into the same model to achieve mutual adjustment. In the analysis of childlessness among post-reproductive women, family owns no property, family support, have health insurance, positive family atmosphere and family social status have been removed because those measures reflect the participant’s current status and less likely to influence their previous reproduction decisions

### Factors associated with reproductive anxiety in childless women of reproductive age

We further investigated the level of reproductive anxiety among childless women of reproductive age (mean = 55.06 on a 0-100 scale, SD = 30.90). As shown in Table 5, at the intrapersonal level, obtaining a Bachelor’s degree or higher was significantly associated with increased reproductive anxiety (β = 7.07, *p* = 0.014). Additionally, greater body image dissatisfaction was also significantly associated with higher reproductive anxiety (β = 4.17, *p* = 0.002). At the interpersonal level, exposure to child abuse during childhood (physical and psychological) was associated with elevated reproductive anxiety (β = 12.41, *p* = 0.006), as was exposure to parental issues during childhood (β = 10.81, *p* = 0.035). At the societal level, women from ethnic minority groups exhibited significantly higher reproductive anxiety compared to their Han ethnic counterparts (β = 11.74, *p* = 0.020). No significant association was detected between other factors and the level of reproductive anxiety (*p* > 0.05, Table 5).

**Table 5:** Factors associated with reproductive anxiety among childless women of reproductive age

	Factors	β	SE	*p* value
Intrapersonal level	Education level (Bachelor’s degree or above)	7.07	2.88	0.014
	Stress coping	-0.06	2.07	0.979
	Body image dissatisfaction	4.17	1.31	0.002
	Risk-taking	-1.50	1.62	0.353
	Subjective philanthropy	1.52	2.45	0.535
Interpersonal level	The only child in the family	1.37	2.71	0.619
	Family owns no property	4.76	3.90	0.224
	Family support	1.09	2.18	0.619
	Exposed to child abuse (physical and psychological)	12.41	4.49	0.006
	Exposed to child abuse (sexual)	-1.29	5.51	0.815
	Exposed to parental issues during childhood	10.81	5.12	0.035
	Witness of intimate partner violence during childhood	-5.84	6.05	0.335
	Intimate partner violence	1.59	1.43	0.268
	Positive family atmosphere	-2.35	2.06	0.255
	Have health insurance	1.73	7.68	0.822
	Family social status	1.86	1.08	0.086
Societal level	Ethnic minority	11.74	5.04	0.020
	Household registration as agricultural population	-1.37	2.78	0.622

Note. SE = standard error. All independent variables were entered into the same model to achieve mutual adjustment

## Discussion

This study is the first large-scale nationwide study in China on the childlessness situation among women and the potential factors associated with childlessness and reproductive anxiety. Fertility patterns in China have undergone significant changes, marked by a notable decline in the proportion of women having three or more children. This shift has been accompanied by a trend toward having one or two children, while the proportion of childless women has gradually increased.

Based on data from a large, representative sample collected in 2023, we found that 10.4% of women of reproductive age were childless, and 3.3% of post-reproductive women were childless throughout their lives. The analysis identified several factors associated with a higher likelihood of childlessness, including obtaining a Bachelor’s degree or higher, being the only child in the family, and exposure to domestic violence, including IPV and childhood abuse. Besides investigating historical and contemporary factors associated with childlessness, exploring the factors associated with reproductive anxiety among childless women can provide insights for policies and interventions aimed at encouraging first-time parenthood to address the issue of low fertility. We found that among the childless women of reproductive age, higher education levels, a higher body image dissatisfaction, exposure to child abuse and parental issues during childhood, and belonging to ethnic minority groups were associated with an elevated level of reproductive anxiety.

Consistent with our findings, Jiang et al. also found that the proportion of childlessness in China has increased markedly from 2010 to 2020, reaching 5.16% for women aged 49 [16]. This is consistent with the conclusion drawn from our descriptive statistics on reproductive structure across all age groups. Although this is a cross-sectional study, the temporal sequence in the associations we observed between potential factors and childlessness or reproductive anxiety can still be established, as most factors occurred before one’s reproductive years, including ethnic group, exposure to child abuse and parental issues during childhood, being the only child in the family, and (to some extent) the education level. The possibility of reverse causality bias would be low in these association estimates.

We consistently observed higher education level as a significant factors in the analyses of both childlessness status and reproductive anxiety, thus providing robust evidence on the education-fertility relationship, despite the inconsistent findings in the previous literature. In line with our findings, many studies have indicated that higher level of women’s education is linked to delayed childbirth or childlessness [24–26]. However, several studies have found an inverse association, suggesting that a higher educational level could potentially increase the feasibility of parenting. In our research context, women with higher education levels may have more knowledge and usage of contraceptives, as well as greater autonomy over reproductive decisions, which increases the likelihood of childlessness [12, 27, 28]. Higher educational attainment also encourages women to actively engage in the competitive labor market, forcing some to make challenging choices between career and marriage or parenthood [27]. 

Interestingly, we found that women who were the only child in the family were more likely to be childless. In line with our observation, two studies in China also showed that individuals from one-child family reported a lower fertility desire or ideal number of children [29, 30]. This phenomenon may be related to family culture or atmosphere, but the explanations still warrant further research. In addition, we observed a positive association between body image dissatisfaction and reproductive anxiety, which aligns with previous findings that suggest a positive body image is associated with greater reproductive intention [31]. Adolescents with body image dissatisfaction have also been identified in existing literature as exhibiting heightened generalized anxiety symptoms [32]. In adulthood, higher levels of body image dissatisfaction correlate with greater concern regarding the potential impact of reproduction on one’s body, thus intensifying reproductive anxiety. Additionally, this study has shown that reproductive anxiety is more pronounced among ethnic minorities in China compared to Han Chinese. Unfortunately, we found little evidence from the previous literature on the ethnicity difference for reproductive anxiety in China. This conclusion warrants further validation and may have policy implications on ethnicity-specific interventions.

Another important finding in our study was the association between various domestic violence variables and childlessness status or reproductive anxiety. It was plausible that early-life experiences of child abuse and parental issues could increase women’s anticipated difficulties or concerns of child-rearing once they reach adulthood or get married, thus increasing their reproductive anxiety. As for the association between exposure to IPV and childlessness status, there could be a bidirectional relationship as infertile women are more likely to suffer from IPV [33]. Future longitudinal studies with repeated measurements are needed to clarify the temporal relationship between these two variables.

This study demonstrates that IPV strongly predicts both childlessness among women of reproductive age and post-reproductive age, underscoring its critical role in shaping fertility outcomes. From a psychological perspective, childhood exposure to abuse—both physical and psychological—emerges as one of the most influential factors among the three levels of factors examined, significantly contributing to reproductive anxiety. These findings highlight the importance of integrating violence prevention into reproductive health policies. Future efforts should focus on understanding how early-life adversity and IPV affect fertility behaviors and mental health. Evidence-based interventions targeting violence against women and children are urgently needed, alongside policy frameworks to address these issues systematically.

Addressing violence is central to strategies for improving societal well-being. This study reveals the intergenerational effects of childhood maltreatment, which continue into adulthood and shape fertility-related psychological outcomes in the next generation. Comprehensive approaches involving mental health support, legal protections, and public education are essential to mitigating these enduring impacts and fostering healthier family environments. Such efforts are crucial to reducing reproductive anxiety and improving fertility outcomes on both individual and societal levels.

There are several limitations in the present study. Due to the cross-sectional design of this study, conclusions cannot be drawn regarding the causality of the observed associations, especially for the contemporary factors such as IPV exposure and body image dissatisfaction. In addition, childlessness is typically categorized into three types [34]: (1) biological incapacity for childbirth, (2) intentional choice of childlessness, and (3) unintentional childlessness (“coincidental childlessness”). Some researchers classify both intentional and unintentional childlessness as voluntary, referring to individuals or couples actively choosing not to have children for diverse reasons [35, 36]. The factors associated with actual childlessness identified in this study should play a more significant role in voluntary childlessness. Finally, several unmeasured factors warrant consideration in the analysis of women’s current childlessness, including age at marriage, reproductive health and fertility capability of women and their partners, as well as experiences of miscarriage and child loss, which were not accounted for in this study.

## Conclusions

Based on the most recent nationwide survey data from China, this study identifies factors associated with childlessness and reproductive anxiety. It underscores the influence of education, family support, and exposure to domestic violence on reproductive outcomes. The findings highlight the need for policies that address domestic violence prevention and provide support for women with a history of childhood adversity. Incorporating these issues into reproductive health policies could help reduce reproductive anxiety and the prevalence of childlessness.

## Electronic supplementary material

Below is the link to the electronic supplementary material.

Supplementary Material 1

## Data Availability

Individual-level data from the 2023 Psychology and Behaviour Investigation of Chinese Residents Survey has not been made publicly available; requests for de-identified data are subject to approval by the corresponding authors.
